# Low Effective Dispersal of Asexual Genotypes in Heterogeneous Landscapes by the Endemic Pathogen *Penicillium marneffei*

**DOI:** 10.1371/journal.ppat.0010020

**Published:** 2005-10-28

**Authors:** Matthew C Fisher, William P Hanage, Sybren de Hoog, Elizabeth Johnson, Michael D Smith, Nicholas J White, Nongnuch Vanittanakom

**Affiliations:** 1 Department of Infectious Disease Epidemiology, St Mary's Hospital Campus, Imperial College London, London, United Kingdom; 2 Centraalbureau voor Schimmelcultures, Utrecht, The Netherlands; 3 Mycology Reference Laboratory, Health Protection Agency, Bristol, United Kingdom; 4 Department of Microbiology, Taunton and Somerset Hospital, Taunton, United Kingdom; 5 Faculty of Tropical Medicine, Mahidol University, Bangkok, Thailand; 6 Department of Microbiology, Faculty of Medicine, Chiang Mai University, Chiang Mai, Thailand; University of California, Berkeley, United States of America

## Abstract

Long-distance dispersal in microbial eukaryotes has been shown to result in the establishment of populations on continental and global scales. Such “ubiquitous dispersal” has been claimed to be a general feature of microbial eukaryotes, homogenising populations over large scales. However, the unprecedented sampling of opportunistic infectious pathogens created by the global AIDS pandemic has revealed that a number of important species exhibit geographic endemicity despite long-distance migration via aerially dispersed spores. One mechanism that might tend to drive such endemicity in the face of aerial dispersal is the evolution of niche-adapted genotypes when sexual reproduction is rare. Dispersal of such asexual physiological “species” will be restricted when natural habitats are heterogeneous, as a consequence of reduced adaptive variation. Using the HIV-associated endemic fungus *Penicillium marneffei* as our model, we measured the distribution of genetic variation over a variety of spatial scales in two host species, humans and bamboo rats. Our results show that, despite widespread aerial dispersal, isolates of *P. marneffei* show extensive spatial genetic structure in both host species at local and country-wide scales. We show that the evolution of the *P. marneffei* genome is overwhelmingly clonal, and that this is perhaps the most asexual fungus yet found. We show that clusters of genotypes are specific to discrete ecological zones and argue that asexuality has led to the evolution of niche-adapted genotypes, and is driving endemicity, by reducing this pathogen's potential to diversify in nature.

## Synopsis

Scientists believe that micro-organisms are spread around the planet on currents of air, a hypothesis that is known as “ubiquitous dispersal”. While fungi release huge quantities of widely dispersed spores, it is not known why many species remain endemic to specific regions around the globe. Research by the authors suggests an answer to this conundrum, by investigating the genetic structure of a fungus, *Penicillium marneffei,* that causes disease in people with damaged immune systems. This research has shown that *P. marneffei* spores can be dispersed over a wide distance, but fail to penetrate the new environments that they find themselves in. This appears to be because the fungus has largely dispensed with sexual reproduction, which means that its ability to adapt to new challenges is limited. The authors use DNA typing to show that different “clones” of the fungus are associated with different environments, and suggest that adaptation to these environments is constraining the organism's ability to successfully disperse in nature. This may explain why *P. marneffei* is endemic to a relatively small area of southeast Asia, and the authors go on to suggest that the long-term consequence of this strategy may be the eventual extinction of the organism.

## Introduction

In eukaryotes, sexual recombination is an almost universal phenomenon [1,2]. However, in many microbial eukaryotes, such as fungi, sexual reproduction is facultative, so loss-of-meiosis mutations are effectively neutral. Such mutations will, on average, be fixed by genetic drift in approximately 2*N_e_* generations (where *N_e_* is the effective population size [3]), leading to the possibility that populations and species may lose sexual competence. Modern ecological opinion holds that populations of microbial eukaryotes are huge owing to continent-wide dispersal via airborne spores; this is known as the “ubiquitous dispersal hypothesis” [4,5]. If this hypothesis is correct, then the large value of *N_e_* for these organisms will negate drift as a significant force in their evolution, and mating competence will be preserved. However, recent studies of aerially dispersed fungi have shown the full continuum of population genetic structures, from global panmixia (e.g., *Aspergillus fumigatus* [6]) through to geographic endemicity and pronounced biogeographic population structure (e.g., *Histoplasma capsulatum* [7], *Coccidioides immitis* [8], *Blastomyces dermatitidis* [9], and *Penicillium marneffei* [10]). To understand why these fungi vary so widely in their ability to undergo successful long-distance aerial dispersal, we need to critically evaluate the ubiquitous dispersal hypothesis.

Theoretical and empirical work has shown that sex acts to increase the rate of adaptation to new environments by increasing the variance in fitness between offspring [11,12]. A corollary of this theory is that we can make a prediction that reduced sexual competence, and hence reduced recombination rates, between species are expected to correlate with a reduced potential to disperse within heterogeneous environments. Loss of sex may therefore explain why endemic species occur in the face of widespread aerial dispersal.

**Figure 1:**
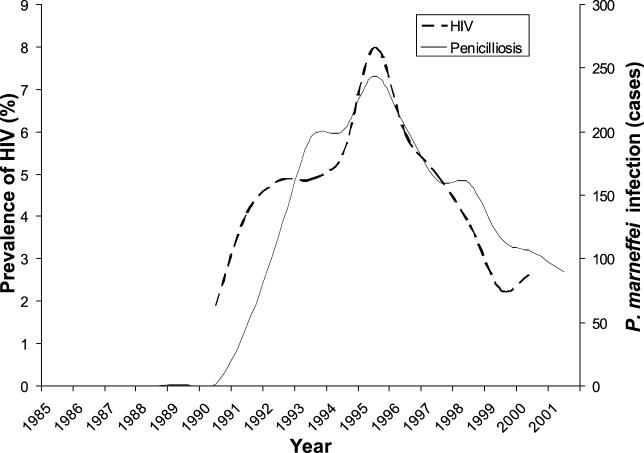
Co-Emergence of AIDS and Human Penicilliosis in Northern Thailand Temporal emergence of HIV (antenatal data, 1990–2000 [38]) and human *P. marneffei*–associated penicilliosis (1985–2001; Maharaj Hospital, Chiang Mai) for the Chiang Mai region, northern Thailand.

We investigated this hypothesis by subjecting the human pathogenic fungus *P. marneffei* to an analysis designed to describe the effects of (i) space, (ii) reproductive mode, and (iii) host on this species's population genetic structure within Thailand. The fungus is endemic to a narrow band of southeast Asia [10] and has a simple haploid lifecycle whereby filamentous soil mycelia give rise to aerially borne conidia [13]. If inhaled, *P. marneffei* disseminates as an intracellular yeast, and, as a consequence, the fungus has recently emerged as a major opportunistic infection of HIV/AIDS patients in this populous region (Figure 1). *P. marneffei* is morphologically asexual and is a member of the estimated one-fifth of fungal species that have no known sexual state. Interestingly, surveys of native southeast Asian rodent populations have shown that *P. marneffei* is associated strongly with bamboo rats, but not other rodent species [14–16]. To test the hypothesis that different lineages of *P. marneffei* may have adapted to infect different hosts, we acquired bamboo rat isolates from several provinces across Thailand. We further acquired isolates of *P. marneffei* from a cohort of AIDS patients from across Thailand and used high-resolution multilocus genotyping to analyse these, and the bamboo rat, isolates.

## Results

### Naturally Occurring Diversity in Isolates from Humans and Bamboo Rats

**Figure 2:**
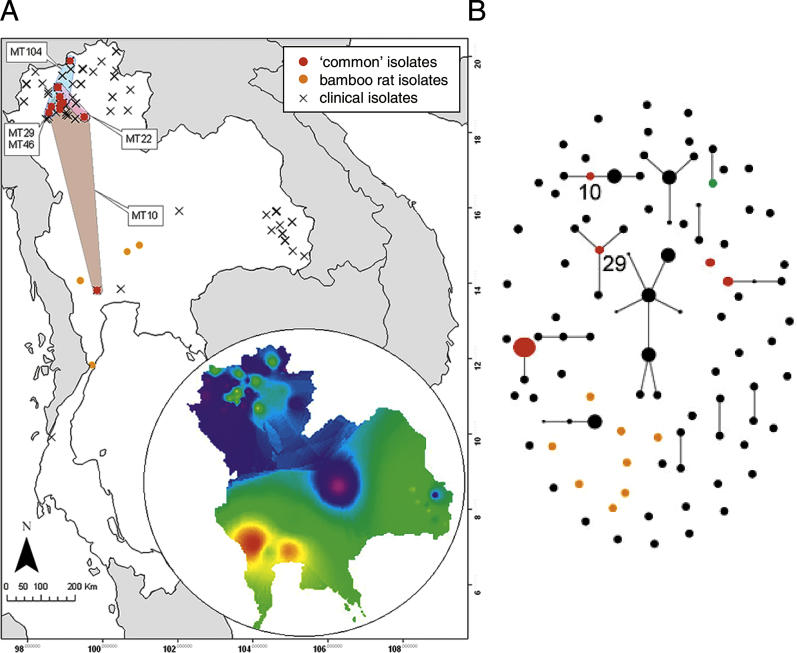
Spatial Distribution of Isolates and Genetic Diversity in Thailand (A) Spatial distribution of sampled isolates in Thailand. Isolates that are found to co-infect both humans and bamboo rats are shown in red, and the clone range of each of these co-infecting MTs is shown as coloured regions. Inset shows the geographic variation of the PC1, which accounts for 18.8% of the total genetic variation. Spatial interpolation of the PC1 values (with colours representing the predicted degree of difference between sample points) illustrates gradients in allele frequencies between northern, eastern, and south-central Thailand. (B) eBURST analysis of the Thailand *P. marneffei* dataset, with isolates coloured as noted in (A). The single green point refers to an isolate that was recovered from soil. Each clone is represented by a point, the area of which is representative of the frequency of the clone. Clones that differ from one another at a single locus, and are thus inferred to be directly related (single locus variants), are joined by lines.

Genetic diversity in our study populations was high and of the 169 isolates subjected to analysis, we were able to assign each isolate into one of 97 unique multilocus genotypes, known as microsatellite types (MTs). Epidemiological connectivity between human and bamboo rats was explored using the program eBURST [17]. This approach searches for groups of related MTs, then ascertains patterns of descent within groups. Under a model of simple asexual reproduction, founding genotypes will rise in frequency over time and subsequently diversify by the accumulation of mutations into a complex of closely related genotypes, known as a clonal complex. Such complexes are identified by eBURST as groups of genotypes that are linked by differing at only one out of the 21 multilocus microsatellite typing (MLMT) loci (known as single locus variants). Analysis of our MLMT dataset by eBURST showed that while 83 MTs were specific to *P. marneffei* that had been recovered from HIV-positive patients, five MTs were found to infect both humans and bamboo rats (Figure 2). This is direct evidence that *P. marneffei* isolates that are capable of infecting both humans and rodents do occur in nature. eBURST identified 13 clonal complexes in our dataset of MLMT genotypes. Of the MTs that are shared between humans and bamboo rats, two (MTs 10 and 29) were designated as the primary founders of the clonal complex within which they occur. For the seven isolates comprising MT 29, six were recovered from the bamboo rat species *Rhizomys sumatrensis,* from two sampling sites in northern Thailand separated by 50 km. These data show that the MT 29 genotype is over-represented in at least two spatially separated rodent populations. This rat-associated founder genotype has diversified into a clonal complex with at least four descendants that are found infecting humans (Figure 2B). Therefore, there is no evidence within our data for fungal lineages that are specific to bamboo rats, and hence no indication that co-evolution is occurring between host and pathogen. In this case, any observed species-specific clustering of genotypes is most likely due to the spatial clustering of genetic diversity in *P. marneffei.*

### Spatial Components of Genetic Diversity

To investigate whether there is a spatial component to *P. marneffei*'s population genetic structure, we calculated a genetic distance matrix for all pairwise combinations of isolates by using an allele-sharing index (*D*_AS_ [18]). This genetic distance is based on allele-frequency data and calculates multilocus pairwise distance measurements as 1 − (the total number of shared alleles at all loci/*n*), where *n* is the number of loci compared. An associated geographic distance matrix was calculated as the distances between the coordinates of each bamboo rat sampling site and/or patient address [19]. Principal components analysis (PCA) was performed on the total matrix of individual genetic distances, and spatial relationships were visualised by plotting associated values in a geographical information system (GIS). This analysis showed that the first principal component (PC1) explains 18.8% of the total observed genetic diversity. We visualised the PC1 values as a continuous surface, by the use of an inverse-distance-weighted kriging procedure (Figure 2A). Kriging is a computational procedure that interpolates between our sample points using least-squares estimation to arrive at a surface of expected values, based on our known principal component values. That our surface shows spatial correlations between PC1 values clearly demonstrates that there is a broad division of genotypes into northern, eastern, and southern populations of *P. marneffei*.

**Figure 3:**
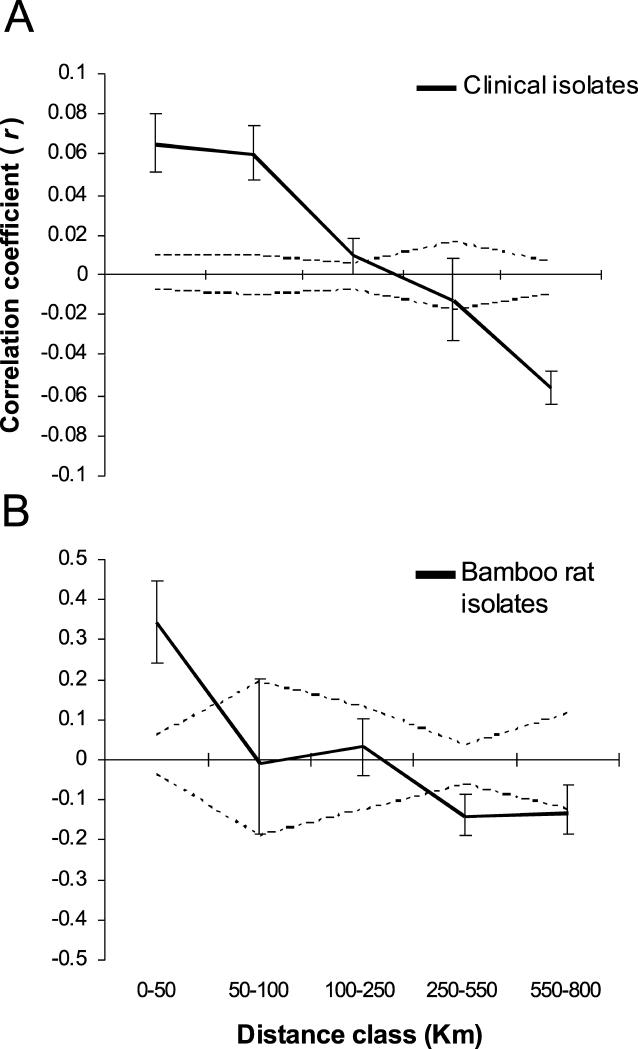
Correlograms Showing the Correlation Coefficient *r* as a Function of Distance for Human Clinical Isolates and Bamboo Rat Isolates Data for (A) human clinical isolates and (B) bamboo rat isolates. Values for *r* are shown as solid lines with 95% CIs calculated by bootstrapping the dataset. Also shown are the 95% CIs around the null hypothesis of randomly distributed genotypes of *P. marneffei,* calculated over 1,000 permutations of the data, shown as dotted lines.

We further investigated this broad spatial pattern by calculating the maximum spatial distance covered by each of the observed *P. marneffei* MTs. This “clone distance” was measured as the geodesic line between the spatial latitudinal and longitudinal coordinates of the most distantly related representatives of each MT. Analysing the spatial distribution of each MT using our GIS showed that 22% of *P. marneffei* MTs were recovered from more than one site, corresponding to an average radial distance of 28.6 km (95% confidence interval [CI] 20.8–36.2), and an average geographic clonal coverage of 1,975 km^2^ (95% CI 1,506–2,444; Table 1). We investigated whether these spatially “patchy” *P. marneffei* clones are randomly dispersed in two-dimensional space by calculating the autocorrelation coefficient, *r,* for all pairwise combinations of individual-by-individual multilocus genetic distances (*D*_AS_) correlated against geographic distance [20]. Isolates that are more related to one another than is expected from a randomly mating population are characterised by a positive value for *r,* and if isolates are spatially structured, then *r* will exhibit a negative correlation with distance. These correlograms confirmed the results from our PCA by showing that there is strong, and significant, positive spatial genetic structure that undergoes rapid decay at greater scales (Figure 3). The autocorrelation coefficient observed between bamboo rat isolates showed that (i) relatedness was significantly higher than that found in clinical isolates at the same local scale (*p* < 0.01) and (ii) the extent of non-random genetic structure was significantly less for bamboo rat isolates (109 km compared to 367 km for human isolates). We interpret these data as showing that the high mobility of humans, relative to bamboo rats, is reflected in an increased probability of infection beyond the scale of the local population of *P. marneffei*.

**Table 1:**
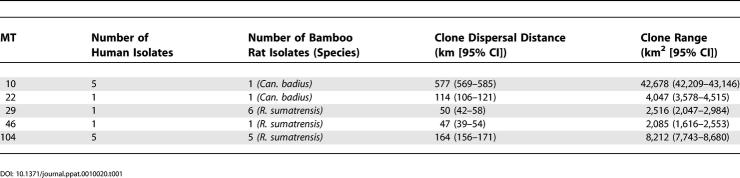
Dispersal Distance and Clone Range for MTs Common to Both Humans and Bamboo Rats

### Relative Contributions of Asexual versus Sexual Reproduction

The occurrence of 13 clonal complexes (see Figure 2B) suggests that there is a significant asexual component to the population genetic structure of *P. marneffei*. We quantified the relative contributions of recombination and mutation to the generation of diversity in this dataset by examining the changes that have generated single locus variants. Changes that incorporate unique alleles are most likely due to mutation and those that utilise pre-existing alleles are most likely due to recombination. In order to prevent our estimate being biased by loci under unusual selective pressures, we excluded the highly diverse or uniform loci from the analysis. Defining recombination and mutation as above, we found that 97% of alleles were found within a single clonal complex. Using this value, we were able to estimate from eBURST that the ratio of recombination to mutation within our dataset was 1:4.7. This value is less than estimates of the same ratio for many bacterial species [21], and is surprisingly clonal for a fungal species. Moreover, our estimate is very conservative, because the mode of mutation in microsatellites by strand-slippage mispairing will inevitably create alleles that are identical by size, but not by descent. As a consequence, a proportion of the changes scored as recombination will in fact be mutations.

To analyse the extent of multilocus linkage disequilibrium, we controlled for the effects of spatial structure by using our GIS to restructure the dataset into populations that comprised at least 20 isolates falling within a single circle of radius 109 km (corresponding to our best estimate for the scale of a local population). This procedure identified two groups of isolates with centres in Chiang Mai and Ubon Ratchathani. We calculated a standardised measure of multilocus disequilibrium, 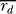 [22], and compared this value to those calculated from a comparable MLMT analysis of two related morphologically asexual fungal pathogens exhibiting endemic distributions, *Coccidioides immitis* and *C. posadasii* [23]. This analysis showed that 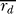 was highly significant for *P. marneffei,* even when we took into account direct clonal reproduction by removing identical genotypes. Interspecific comparisons of 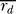 showed that *P. marneffei* was significantly more asexual than either of the two *Coccidioides* species (Table 2). As an additional test of the hypothesis of clonality, a maximum parsimony approach was used to find the shortest tree that fitted the *P. marneffei* data, then randomisation protocols were used to simulate 1,000 randomly mating populations. In all cases, the observed tree was significantly shorter than the null distributions, demonstrating significant structure in our data (Figure S1). As we have controlled for spatial population structure by including only isolates from a small geographic area, and have considered them together with the evidence from the eBURST analysis, the most likely cause of this observed multilocus genetic disequilibrium is evolution by clonal descent.

**Table 2:**
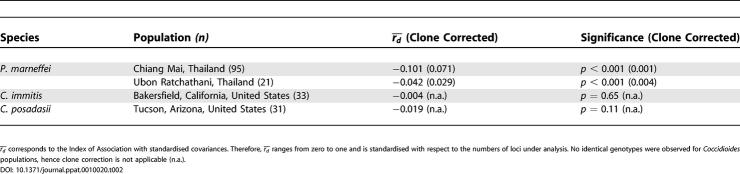
Multilocus Linkage Disequilibrium for *P. marneffei* and *Coccidioides* spp.

### Associations between Genetic Diversity and Ecological Heterogeneity

It is clear from the above analyses that the population genetic structure of *P. marneffei* has two components: one caused by a low effective rate of dispersal and the other by a low effective rate of recombination. The adaptation of *P. marneffei* to an aerial mode of transmission, manifested by its ability to produce essentially unlimited infectious conidia, in the same size range as microbes (2–3 μm), presents a conundrum. *P. marneffei* is highly abundant, as is shown by the high prevalence of infection in both human and bamboo rat populations in endemic areas. Furthermore, widespread dispersal evidently occurs, as we have been able to demonstrate infection of humans and bamboo rats with identical clonal genotypes over a 47- to 577-km range (Table 1). However, this dispersive capacity has not been translated into widespread gene flow, and significant population genetic structure occurs.

Under neutral community models [24], differences in geographic biodiversity are attributable primarily to differences in dispersal rate. However, if the relative fitness of genotypes varies between regions as a consequence of strong disruptive selection, then locally adapted genotypes will tend to outcompete any incoming immigrants even in the face of frequent dispersal. A key parameter that will therefore determine the relative fitness of isolates between different regions is the strength of local selection, i.e., the degree of environmental heterogeneity. We tested whether environmental heterogeneity correlates with genetic diversity by performing ecological niche modelling based on our primary occurrence data using Genetic Algorithms for Rule-Set Prediction (GARP [25]). This algorithm uses digital maps to summarise environmental variables, then identifies associations between point occurrence data and their ecological dimensions. Rule sets that define a niche with the highest predictive accuracy (based on known occurrences) are subsequently used to generate digital maps with areas of predicted niche presence and absence (for details see [26]).

**Figure 4:**
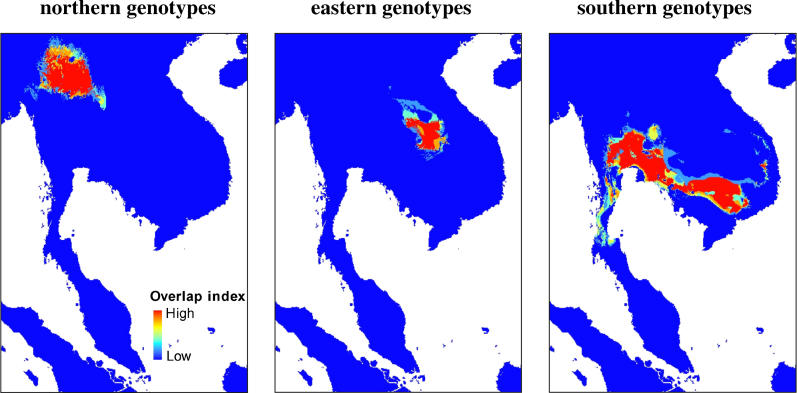
Predicted Distributions for Three Classes of Genotype: Northern, Eastern, and Southern Thailand The “overlap index” represents the synthesised output from 20 optimal GARP models. See Figure S2 for data sources.

We modelled southeast Asia's ecology using the BIOCLIM 2.5 minute dataset [27], the Food and Agricultural Organization digital soil map, and remotely sensed digital vegetation maps of southeast Asia with a 1-km^2^ resolution (Figure S2). Three categories of genotype were used as occurrence points, corresponding to the northern, eastern, and southern Thailand genotypes determined by PCA (see Figure 2A, inset). Projecting the rule sets from these analyses clearly illustrates non-overlapping predictions for the distributions of the three genotype classes (Figure 4). This analysis shows that environmental heterogeneity exists within Thailand at the scales investigated here, and that there is a clear association between genetic and ecological diversity. The rule sets postulate a wider distribution for the southern genotype class, as the genotypes in this class are associated with a set of environmental variables that are more widely distributed than is found associated with the northern and eastern Thailand genotypes. Interestingly, the southern Thailand genotypes are predicted to occur within Viet Nam, a hypothesis that should be examined as Viet Nam is currently undergoing an emergence of human penicilliosis in association with the epidemic of HIV within this region.

## Discussion

Genuinely asexual fungi are rare and few have passed the population genetic tests that we have presented here; the genetic footprint of recombination appears to be ubiquitous in nature [28]. While we are not ruling out the presence of recombination in *P. marneffei,* the low frequencies that we have measured will tend to decrease additive fitness variance within populations [1], leading to the evolution of a “rugged” fitness landscape with multiple peaks and valleys. In a heterogeneous environment, variance in fitness between clonal lineages is expected to lead to populations that are adapted to local conditions [11,12]. In this case limited dispersal then becomes an inevitable consequence of asexuality, as invading clones are outcompeted by better adapted local conspecific competitors because of the decreased ability of invaders to diversify within novel environments [11]. In such a system, spatial metapopulation structure will further increase biases in the frequencies of mating-types and loss-of-meiosis mutations by increasing the rate of genetic drift. Eventually, *N_e_* is expected to become depressed to the extent that mating competence within populations will be irrevocably lost [29,30]. Our work shows that *P. marneffei* is perhaps the most asexual fungus yet found. We provide a theoretical mechanism to account for *P. marneffei*'s extreme geographic endemism and fragmented population genetic structure, by arguing that the clonality we observe is driving niche adaptation in a heterogeneous environment. Further, our conclusions clearly falsify the ubiquitous dispersal hypothesis for *P. marneffei,* and data on the population genetic structure of other endemic fungi, as well as bacteria, suggest that our results will have broader relevance to microbial systems [28,31,32]. Further tests of our hypothesis will rely on experimental measurements of the relative fitness of asexual and sexual fungal lineages in controlled, heterogeneous environmental backgrounds. Such investigations will ultimately show whether microbial endemicity is a signpost of genomic decay, heralding the onset of a species's eventual extinction.

## Materials and Methods

*P. marneffei* isolates were acquired as cultures from HIV/AIDS patients who were attending regional central hospitals in Chiang Mai (northern Thailand), Ubon Ratchathani (eastern Thailand), and Bangkok (south-central Thailand). Isolates were georeferenced with latitudinal and longitudinal coordinates corresponding to the recorded patient address. Our study obtained 146 epidemiologically unlinked human isolates of *P. marneffei* from patients widely distributed across Thailand (see Figure 2A). We further acquired 23 isolates from three species of bamboo rat, *Rhizomys pruinosus, R. sumatrensis,* and *Cannomys badius;* these isolates had all been found infecting the liver and spleen of healthy adult rodents [14,15]. Isolates were grown on slants containing Sabouraud's agar medium. Following homogenisation of mycelia, DNA was extracted using a Qiagen (Valencia, California, United States) DNeasy Tissue kit according to the manufacturer's instructions. The purified DNA was then used as a template to amplify 21 microsatellite loci using fluorescently labelled primers and a PCR multiplexing protocol [33]. Subsequently, PCR products were visualised on an Applied Biosystems (Foster City, California, United States) model 3700 capillary sequencer and the alleles were scored using Genotyper software (Applied Biosystems). All genotypes were assigned a specific MT identifier according to the *P. marneffei* MLMT scheme [34,35]; associated data and the MTs are accessible via the Internet at http://pmarneffei.multilocus.net/ (Table S1).

## Supporting Information

Figure S1 Distribution of Tree Lengths for Northern Thailand Using the program PAUP* 4.0, maximum parsimony was used to find the shortest tree(s) that fitted the data. In order to test whether the observed data contained a greater phylogenetic signal when compared to sexual populations, the datasets were artificially recombined 1,000 times, and the lengths of their most parsimonious trees were compared to those found for the observed dataset. This process was repeated for datasets in which all identical genotypes had been removed (clone-corrected data).
(84 KB PPT)

Figure S2 Point Occurrence Data and Environmental Layers Used in GARP Analysis GARP searches for non-random associations between points of known occurrence compared to the overall study region (in this case southeast Asia). The algorithm selects optimal models after a number of iterations, based on a training subset of the data, then uses the remaining data to test model quality. The routines used here were run in Openmodeller (http://openmodeller.sourceforge.net/), and the output visualised using ArcMap 8.3 (ESRI, Redlands, California, United States). Three categories of genotype were used as occurrence points, corresponding to the northern, eastern, and southern Thailand genotypes determined by PCA (see Figure 1). Because our data are address-based, and therefore only corresponds to an estimate of where the patient (or bamboo rat) was infected, we resampled each environment within an area for a circle of radius 28.6 km (corresponding to our estimate of clone dispersal distance) around the coordinate of our point sample. This was repeated 20 times for each sample point, resulting in the point distributions seen in maps 1–3. Twenty-one digital environmental layers were obtained (above). These correspond to the Food and Agricultural Organization's *Digital Soil Map of the World and Derived Soil Properties* [36] (map 4), vegetation cover and classification, derived from the SPOT-4 remote sensor [37] (map 5), and 2.5 minute WORLDCLIM layers [29] (maps 6–24), corresponding to annual mean temperature (map 6), mean diurnal range (mean of monthly [maximum temperature − minimum temperature]) (map 7); isothermality (map 8), temperature seasonality (standard deviation × 100) (map 9), maximum temperature of warmest month (map 10), minimum temperature of coldest month (map 11), temperature annual range (map 12), mean temperature of wettest quarter (map 13), mean temperature of driest quarter (map 14), mean temperature of warmest quarter (map 15), mean temperature of coldest quarter (map 16), annual precipitation (map 17), precipitation of wettest month (map 18), precipitation of driest month (map 19), precipitation seasonality (coefficient of variation) (map 20), precipitation of wettest quarter (map 21), precipitation of driest quarter (map 22), precipitation of warmest quarter (map 23), and precipitation of coldest quarter (map 24).
(507 KB PPT)

Table S1 MLMT Allele Summary for 169 *P. marneffei* Isolates (18 KB PDF)

## Abbreviations

CI: confidence interval
GARP: Genetic Algorithms for Rule-Set Prediction
GIS: geographical information system
MLMT: multilocus microsatellite typing
MT: microsatellite type
PC1: first principle component
PCA: principal components analysis
