# Supplementary material for: Low Effective Dispersal of Asexual Genotypes in Heterogeneous Landscapes by the Endemic Pathogen Penicillium marneffei
Source: PLoS Pathog. 2005 Oct 28;1(2):e20. doi: 10.1371/journal.ppat.0010020 (PMC1266309; doi:10.1371/journal.ppat.0010020)
Supplement: Figure S1 — Using the program PAUP* 4.0, maximum parsimony was used to find the shortest tree(s) that fitted the data. In order to test whether the observed data contained a greater phylogenetic signal when compared to sexual populations, the datasets were artificially recombined 1,000 times, and the lengths of their most parsimonious trees were compared to those found for the observed dataset. This process was repeated for datasets in which all identical genotypes had been removed (clone-corrected data). (84 KB PPT) [file ppat.0010020.sg001.ppt]

## Slide 1
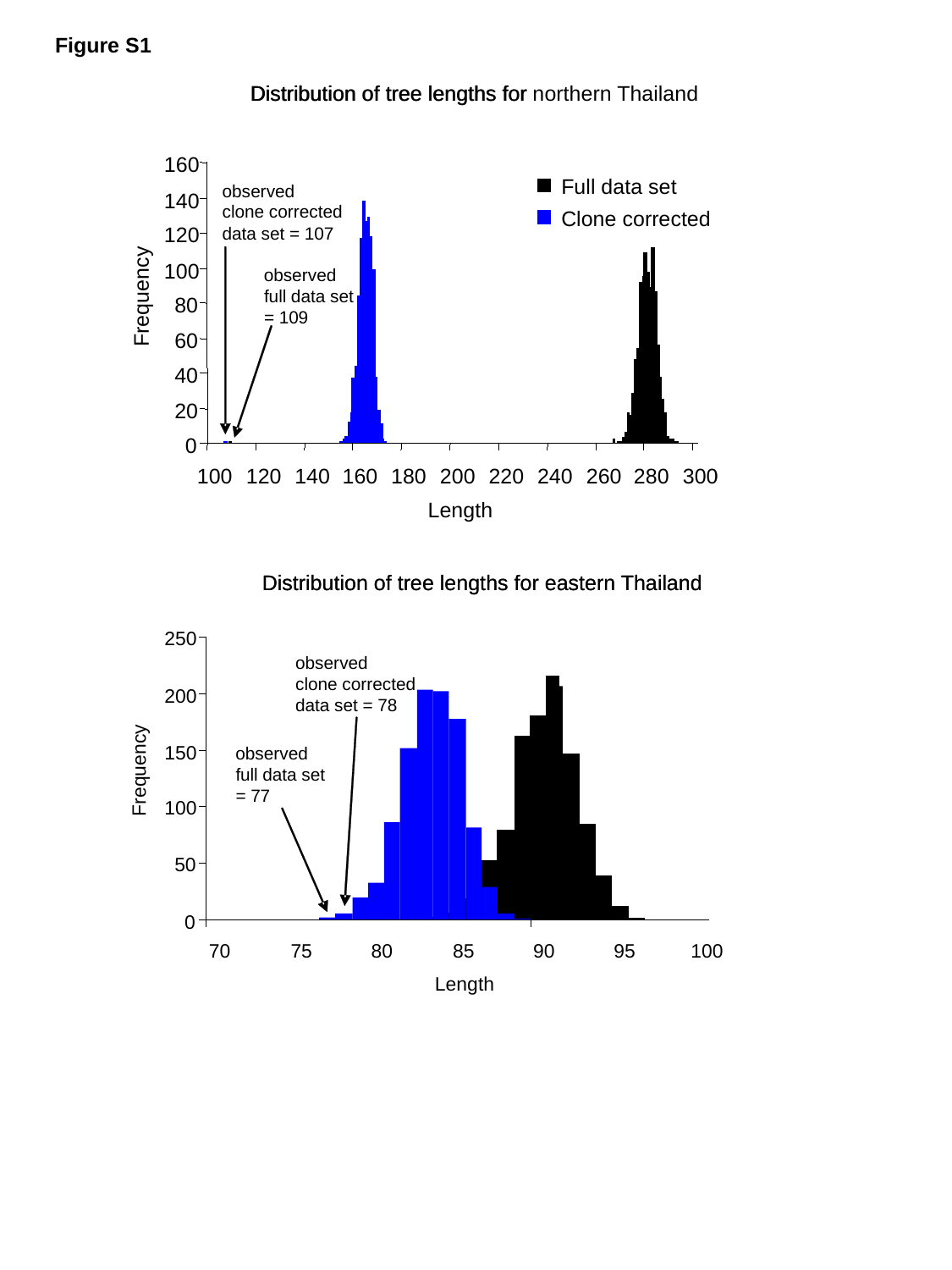

Figure S1
Distribution of tree lengths for northern Thailand
Distribution of tree lengths for
160
Full data set
observed
140
clone corrected
Clone corrected
120
data set = 107
100
observed
Frequency
full data set
80
= 109
60
40
20
0
100
120
140
160
180
200
220
240
260
280
300
Length
Distribution of tree lengths for eastern Thailand
250
Full data set
observed
Clone corrected
clone corrected
200
data set = 78
150
observed
Frequency
full data set
= 77
100
50
0
70
75
80
85
90
95
100
Length
160
Full data set
observed
140
clone corrected
Clone corrected
120
data set = 107
100
observed
Frequency
full data set
80
= 109
60
40
20
0
100
120
140
160
180
200
220
240
260
280
300
Length
Distribution of tree lengths for eastern Thailand
250
Full data set
observed
Clone corrected
clone corrected
200
data set = 78
150
observed
Frequency
full data set
= 77
100
50
0
70
75
80
85
90
95
100
Length
250
Full data set
observed
Clone corrected
clone corrected
200
data set = 78
150
observed
Frequency
full data set
= 77
100
50
0
70
75
80
85
90
95
100
Length
