# Supplementary figures and images for: Low Effective Dispersal of Asexual Genotypes in Heterogeneous Landscapes by the Endemic Pathogen Penicillium marneffei
Source: PLoS Pathog. 2005 Oct 28;1(2):e20. doi: 10.1371/journal.ppat.0010020 (PMC1266309; doi:10.1371/journal.ppat.0010020)

## Slide 1
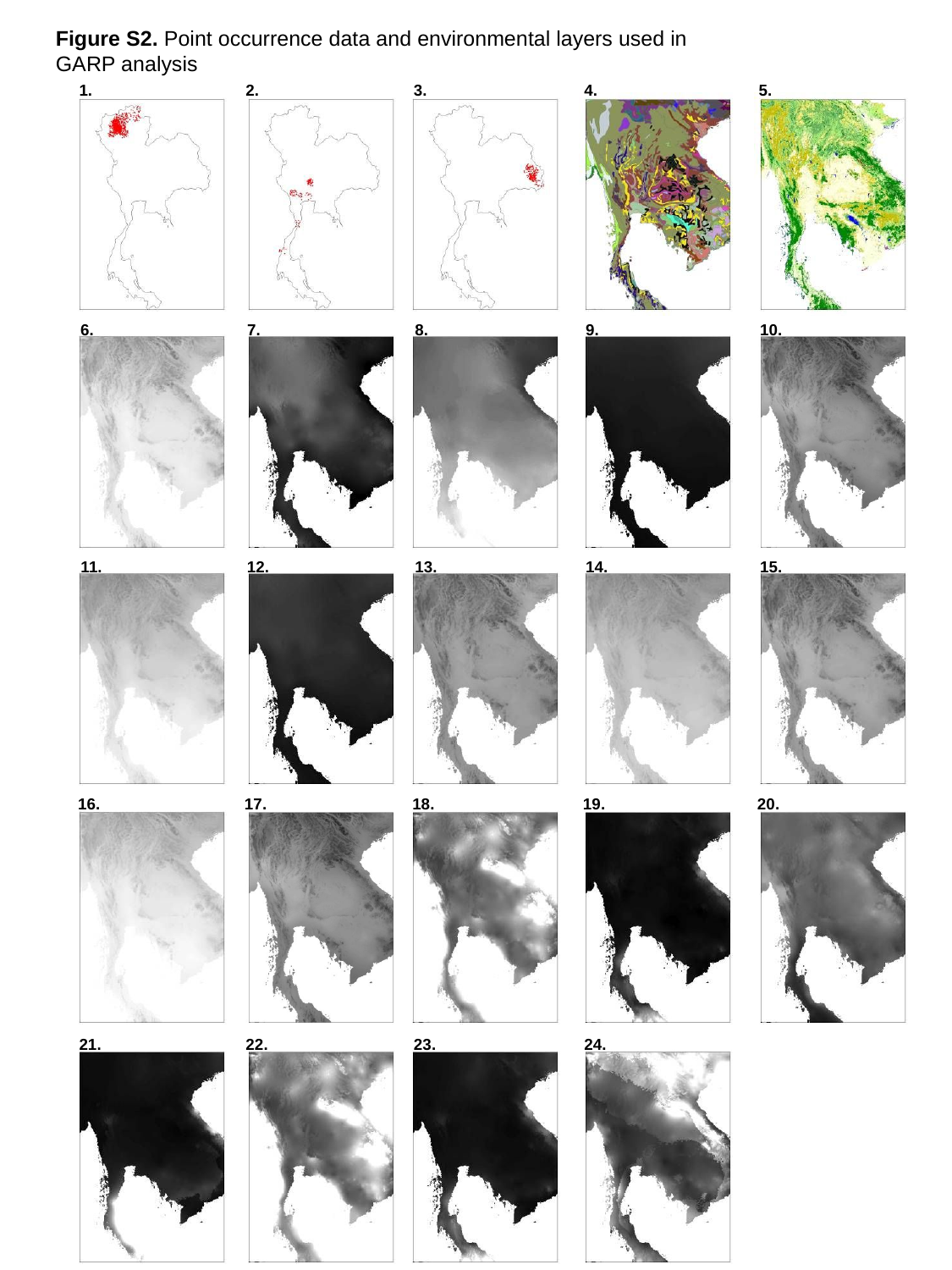

Figure S2. Point occurrence data and environmental layers used in
GARP analysis
1.
2.
3.
4.
5.
6.
7.
8.
9.
10.
11.
12.
13.
14.
15.
16.
17.
18.
19.
20.
21.
22.
23.
24.

Supplement: Figure S2 — GARP searches for non-random associations between points of known occurrence compared to the overall study region (in this case southeast Asia). The algorithm selects optimal models after a number of iterations, based on a training subset of the data, then uses the remaining data to test model quality. The routines used here were run in Openmodeller (http://openmodeller.sourceforge.net/), and the output visualised using ArcMap 8.3 (ESRI, Redlands, California, United States). Three categories of genotype were used as occurrence points, corresponding to the northern, eastern, and southern Thailand genotypes determined by PCA (see Figure 1). Because our data are address-based, and therefore only corresponds to an estimate of where the patient (or bamboo rat) was infected, we resampled each environment within an area for a circle of radius 28.6 km (corresponding to our estimate of clone dispersal distance) around the coordinate of our point sample. This was repeated 20 times for each sample point, resulting in the point distributions seen in maps 1–3. Twenty-one digital environmental layers were obtained (above). These correspond to the Food and Agricultural Organization's Digital Soil Map of the World and Derived Soil Properties [36] (map 4), vegetation cover and classification, derived from the SPOT-4 remote sensor [37] (map 5), and 2.5 minute WORLDCLIM layers [29] (maps 6–24), corresponding to annual mean temperature (map 6), mean diurnal range (mean of monthly [maximum temperature − minimum temperature]) (map 7); isothermality (map 8), temperature seasonality (standard deviation × 100) (map 9), maximum temperature of warmest month (map 10), minimum temperature of coldest month (map 11), temperature annual range (map 12), mean temperature of wettest quarter (map 13), mean temperature of driest quarter (map 14), mean temperature of warmest quarter (map 15), mean temperature of coldest quarter (map 16), annual precipitation (map 17), precipitation of wettest month (map [file ppat.0010020.sg002.ppt]
