# Supplementary material for: Low Effective Dispersal of Asexual Genotypes in Heterogeneous Landscapes by the Endemic Pathogen Penicillium marneffei
Source: PLoS Pathog. 2005 Oct 28;1(2):e20. doi: 10.1371/journal.ppat.0010020 (PMC1266309; doi:10.1371/journal.ppat.0010020)
Supplement: Table S1 — (18 KB PDF) [file ppat.0010020.st001.pdf]

Supplementary Table 1. MLMT allele summary for 169 *Penicillium marneffei* isolates.

Host	Name	MT	Locus																				
			PM22	PM4	PM23	PM6	PM7	PM8	PM9	PM10	PM11	PM12	PM14	PM15	PM17	PM19	PM20	PM21	PM1	PM5	PM24	PM25	PM26
	45P11-49	3	2	1	2	1	7	7	1	2	5	6	4	3	2	4	2	2	2	4	3	2	1
	4208	5	5	2	1	4	8	7	2	2	3	6	4	2	1	6	2	2	3	4	1	3	1
	46P11-10	8	2	1	3	1	6	3	1	2	4	6	5	3	1	3	2	2	3	3	2	3	1
	46P11-12	9	2	1	3	1	6	3	1	2	4	6	5	3	1	4	2	2	3	3	2	3	1
	45P11-32	10	2	3	2	5	4	5	1	2	5	4	4	3	1	3	1	2	3	1	3	3	1
	46P11-25	10	2	3	2	5	4	5	1	2	5	4	4	3	1	3	1	2	3	1	3	3	1
	46P11-43	10	2	3	2	5	4	5	1	2	5	4	4	3	1	3	1	2	3	1	3	3	1
	47P11-3	10	2	3	2	5	4	5	1	2	5	4	4	3	1	3	1	2	3	1	3	3	1
	47P11-5	10	2	3	2	5	4	5	1	2	5	4	4	3	1	3	1	2	3	1	3	3	1
	47P11-8	11	2	3	2	5	4	6	1	2	5	4	4	3	1	3	1	2	3	1	3	3	1
	47P11-9	11	2	3	2	5	4	6	1	2	5	4	4	3	1	3	1	2	3	1	3	3	1
	46P11-3	12	2	3	2	5	4	3	1	2	5	4	4	3	1	4	1	2	3	1	3	3	1
	45P11-40	13	2	3	2	5	4	5	1	2	5	4	4	3	1	4	1	2	3	1	3	3	1
	46P11-1	13	2	3	2	5	4	5	1	2	5	4	4	3	1	4	1	2	3	1	3	3	1
	46P11-11	13	2	3	2	5	4	5	1	2	5	4	4	3	1	4	1	2	3	1	3	3	1
	45P11-6	14	2	3	2	4	3	10	1	1	5	3	3	3	1	4	2	2	3	1	3	3	1
	426	15	2	3	2	4	4	10	1	1	5	3	4	3	1	5	2	2	4	2	3	3	1
	46P11-42	16	2	3	2	5	4	10	1	1	5	3	4	3	1	5	2	2	5	2	3	3	1
	46P11-45	16	2	3	2	5	4	10	1	1	5	3	4	3	1	5	2	2	5	2	3	3	1
	46P11-36	18	2	2	3	1	7	6	1	2	4	6	4	3	2	4	2	2	2	3	3	3	1
	46P11-71	18	2	2	3	1	7	6	1	2	4	6	4	3	2	4	2	2	2	3	3	3	1
	46P11-29	19	2	2	3	1	7	6	1	2	4	6	4	3	2	4	2	3	2	3	3	3	1
	46P11-63	21	2	1	3	1	6	3	1	2	4	6	5	3	1	4	2	2	3	3	3	3	1
	46P11-57	21	2	1	3	1	6	3	1	2	4	6	5	3	1	4	2	2	3	3	3	3	1
	533	22	2	2	2	1	7	3	1	2	4	6	1	3	2	4	2	2	3	3	3	3	1
	45P11-45	23	2	1	2	1	6	3	1	2	4	6	4	3	2	4	2	2	3	3	3	3	1
	496 Y	23	2	1	2	1	6	3	1	2	4	6	4	3	2	4	2	2	3	3	3	3	1
	46P11-61	25	2	2	3	1	6	6	1	2	4	6	4	3	2	4	2	2	3	3	3	3	1
	46P11-64	25	2	2	3	1	6	6	1	2	4	6	4	3	2	4	2	2	3	3	3	3	1
	46P11-59	26	2	1	3	1	7	6	1	2	4	6	4	3	2	4	2	2	3	3	3	3	1
	229_NV	26	2	1	3	1	7	6	1	2	4	6	4	3	2	4	2	2	3	3	3	3	1
	494	26	2	1	3	1	7	6	1	2	4	6	4	3	2	4	2	2	3	3	3	3	1
	46P11-4	29	2	1	2	1	6	3	1	2	4	6	5	3	2	4	2	2	3	3	3	3	1
	45P11-14	30	2	1	2	1	7	3	1	2	4	6	5	3	2	4	2	2	3	3	3	3	1
	45P11-47	31	2	1	2	1	6	5	1	2	4	6	5	3	2	4	2	2	3	3	3	3	1
	46P11-32	32	2	2	2	2	8	6	1	2	4	6	5	3	2	4	2	2	3	3	3	3	1
	46P11-33	32	2	2	2	2	8	6	1	2	4	6	5	3	2	4	2	2	3	3	3	3	1
	46P11-26	33	2	1	2	1	6	6	1	2	5	6	5	3	2	4	2	2	3	3	3	3	1
	389	33	2	1	2	1	6	6	1	2	5	6	5	3	2	4	2	2	3	3	3	3	1
	46P11-67	34	2	1	3	1	6	3	1	2	4	6	6	3	2	4	2	2	3	3	3	3	1
	46P11-58	34	2	1	3	1	6	3	1	2	4	6	6	3	2	4	2	2	3	3	3	3	1
	46P11-70	34	2	1	3	1	6	3	1	2	4	6	6	3	2	4	2	2	3	3	3	3	1
	45P11-23	35	2	1	3	1	7	3	1	2	4	6	6	3	2	4	2	2	3	3	3	3	1
	45P11-15	36	2	1	3	1	6	5	1	2	4	6	6	3	2	4	2	2	3	3	3	3	1
	46P11-22	36	2	1	3	1	6	5	1	2	4	6	6	3	2	4	2	2	3	3	3	3	1
	46P11-24	36	2	1	3	1	6	5	1	2	4	6	6	3	2	4	2	2	3	3	3	3	1
	47P11-1	37	2	2	3	1	6	6	1	2	4	6	5	3	2	5	2	2	3	3	3	3	1
	4174	38	5	3	2	3	7	7	1	1	4	6	3	3	1	8	2	2	3	3	3	3	1
	4182	40	5	3	1	3	7	14	2	2	3	6	3	3	1	10	2	2	3	3	3	3	1
	46P11-65	42	2	1	3	1	7	7	1	2	4	6	4	3	1	4	2	2	4	3	3	3	1
	46P11-66	42	2	1	3	1	7	7	1	2	4	6	4	3	1	4	2	2	4	3	3	3	1
	46P11-15	43	2	2	3	1	7	3	1	2	5	6	4	3	1	4	2	2	4	3	3	3	1
	502 H (Y)	43	2	2	3	1	7	3	1	2	5	6	4	3	1	4	2	2	4	3	3	3	1
	46P11-69	44	2	1	3	1	7	5	1	2	5	6	4	3	2	4	2	2	4	3	3	3	1
	46P11-74	44	2	1	3	1	7	5	1	2	5	6	4	3	2	4	2	2	4	3	3	3	1
	46P11-16	45	2	1	3	1	7	9	1	2	5	6	4	3	2	4	2	2	4	3	3	3	1
	45P11-20	46	2	1	3	1	6	3	1	2	4	6	6	3	2	4	2	2	4	3	3	3	1
	45P11-13	47	2	2	2	1	4	3	1	2	5	4	5	3	2	4	2	2	1	4	3	3	1
	45P11-34	48	2	3	2	1	5	3	1	2	5	4	5	3	2	4	2	2	1	4	3	3	1
	45P11-50	49	2	1	3	1	4	2	1	2	5	4	4	3	2	4	1	2	2	4	3	3	1
	46P11-34	52	2	1	2	1	7	8	1	2	5	6	4	2	2	4	2	2	2	4	3	3	1
	475	55	2	2	2	1	1	3	1	2	5	4	4	3	2	4	2	2	2	4	3	3	1
	45P11-21	56	2	1	2	1	6	7	1	2	5	6	4	3	2	4	2	2	2	4	3	3	1
	46P11-6	56	2	1	2	1	6	7	1	2	5	6	4	3	2	4	2	2	2	4	3	3	1
	46P11-17	56	2	1	2	1	6	7	1	2	5	6	4	3	2	4	2	2	2	4	3	3	1
	46P11-75	56	2	1	2	1	6	7	1	2	5	6	4	3	2	4	2	2	2	4	3	3	1
	45P11-16	57	2	1	2	1	7	7	1	2	5	6	4	3	2	4	2	2	2	4	3	3	1
	45P11-19	57	2	1	2	1	7	7	1	2	5	6	4	3	2	4	2	2	2	4	3	3	1
	45P11-41	57	2	1	2	1	7	7	1	2	5	6	4	3	2	4	2	2	2	4	3	3	1
	46P11-76	57	2	1	2	1	7	7	1	2	5	6	4	3	2	4	2	2	2	4	3	3	1
	47P11-11	57	2	1	2	1	7	7	1	2	5	6	4	3	2	4	2	2	2	4	3	3	1
	45P11-4	58	2	1	2	1	7	8	1	2	5	6	4	3	2	4	2	2	2	4	3	3	1
Human	45P11-7	58	2	1	2	1	7	8	1	2	5	6	4	3	2	4	2	2	2	4	3	3	1
	45P11-48	58	2	1	2	1	7	8	1	2	5	6	4	3	2	4	2	2	2	4	3	3	1
	46P11-35	58	2	1	2	1	7	8	1	2	5	6	4	3	2	4	2	2	2	4	3	3	1
	46P11-56	58	2	1	2	1	7	8	1	2	5	6	4	3	2	4	2	2	2	4	3	3	1
	45P11-33	59	2	1	2	1	8	8	1	2	5	6	4	3	2	4	2	2	2	4	3	3	1
	46P11-18	59	2	1	2	1	8	8	1	2	5	6	4	3	2	4	2	2	2	4	3	3	1
	45P11-12	60	2	1	2	1	7	9	1	2	5	6	4	3	2	4	2	2	2	4	3	3	1
	45P11-30	60	2	1	2	1	7	9	1	2	5	6	4	3	2	4	2	2	2	4	3	3	1
	518	61	2	1	3	1	4	2	1	2	5	4	5	3	2	4	2	2	2	4	3	3	1
	46P11-5	62	2	2	2	1	4	3	1	2	5	4	5	3	2	4	2	2	2	4	3	3	1
	46P11-37	62	2	2	2	1	4	3	1	2	5	4	5	3	2	4	2	2	2	4	3	3	1
	46P11-39	62	2	2	2	1	4	3	1	2	5	4	5	3	2	4	2	2	2	4	3	3	1
	495	62	2	2	2	1	4	3	1	2	5	4	5	3	2	4	2	2	2	4	3	3	1
	46P11-77	63	2	2	2	1	5	3	1	2	5	4	5	3	2	4	2	2	2	4	3	3	1
	46P11-78	63	2	2	2	1	5	3	1	2	5	4	5	3	2	4	2	2	2	4	3	3	1
	46P11-80	63	2	2	2	1	5	3	1	2	5	4	5	3	2	4	2	2	2	4	3	3	1
	332	63	2	2	2	1	5	3	1	2	5	4	5	3	2	4	2	2	2	4	3	3	1
	490	63	2	2	2	1	5	3	1	2	5	4	5	3	2	4	2	2	2	4	3	3	1
	515	63	2	2	2	1	5	3	1	2	5	4	5	3	2	4	2	2	2	4	3	3	1
	511	64	2	2	2	1	4	5	1	2	5	4	5	3	3	4	2	2	2	4	3	3	1
	46P11-13	65	2	1	2	1	7	7	1	2	5	6	4	3	2	5	2	2	2	4	3	3	1
	4179	67	5	3	2	3	7	9	2	2	4	6	3	3	2	8	2	2	2	4	3	3	1
	45P11-10	71	2	1	1	1	4	6	1	1	5	6	3	3	1	4	1	2	3	4	3	3	1
	4177	73	2	2	2	1	6	3	1	2	4	6	3	4	2	4	1	2	3	4	3	3	1
	45P11-31	74	3	1	2	1	4	7	1	1	5	4	4	3	1	5	1	2	3	4	3	3	1
	46P11-31	76	2	1	1	1	7	8	2	2	4	6	6	3	3	12	1	2	3	4	3	3	1
	46P11-68	76	2	1	1	1	7	8	2	2	4	6	6	3	3	12	1	2	3	4	3	3	1
	4204	77	2	3	1	3	2	5	3	2	3	2	4	3	1	1	2	2	3	4	3	3	1
	534	80	3	1	2	1	6	7	1	2	5	6	5	3	1	3	2	2	3	4	3	3	1
	45P11-46	82	2	1	2	1	6	7	1	2	5	6	5	3	1	4	2	2	3	4	3	3	1
	391 HY	86	2	1	3	1	6	8	1	2	5	6	4	3	2	4	2	2	3	4	3	3	1
	46P11-60	87	2	1	3	1	6	2	1	2	5	1	5	3	2	4	2	2	3	4	3	3	1
	45P11-18	89	2	2	3	1	6	3	1	2	4	6	6	3	2	4	2	2	3	4	3	3	1
	46P11-27	89	2	2	3	1	6	3	1	2	4	6	6	3	2	4	2	2	3	4	3	3	1
	4197	92	2	2	2	1	7	6	1	2	4	6	5	3	2	6	2	2	3	4	3	3	1
	4207	93	5	3	2	3	6	7	1	1	4	6	3	3	1	8	2	2	3	4	3	3	1
	46P11-44	96	1	1	2	1	7	8	1	2	4	6	5	3	3	11	2	2	3	4	3	3	1
	47P11-2	97	1	1	2	1	8	8	1	2	4	6	5	3	3	11	2	2	3	4	3	3	1
	47P11-6	97	1	1	2	1	8	8	1	2	4	6	5	3	3	11	2	2	3	4	3	3	1
	47P11-10	98	1	1	2	1	7	7	1	3	4	6	5	3	3	11	2	2	3	4	3	3	1
	517 Y	98	1	1	2	1	7	7	1	3	4	6	5	3	3	11	2	2	3	4	3	3	1
	46P11-38	99	1	2	2	1	7	6	1	2	4	6	5	3	2	12	2	2	3	4	3	3	1
	46P11-62	99	1	2	2	1	7	6	1	2	4	6	5	3	2	12	2	2	3	4	3	3	1
	47P11-7	100	1	2	2	1	7	6	2	2	4	6	5	3	2	12	2	2	3	4	3	3	1
	444	101	1	2	2	1	7	6	1	2	4	6	5	3	2	13	2	2	3	4	3	3	1
	497 Y	102	1	2	2	1	6	6	1	2	4	6	5	3	2	14	2	2	3	4	3	3	1
	45P11-27	104	2	1	2	1	4	3	1	2	5	6	6	3	2	4	2	1	4	4	3	3	1
	46P11-28	104	2	1	2	1	4	3	1	2	5	6	6	3	2	4	2	1	4	4	3	3	1
	46P11-79	104	2	1	2	1	4	3	1	2	5	6	6	3	2	4	2	1	4	4	3	3	1
	46P11-107	104	2	1	2	1	4	3	1	2	5	6	6	3	2	4	2	1	4	4	3	3	1
	531	104	2	1	2	1	4	3	1	2	5	6	6	3	2	4	2	1	4	4	3	3	1
	45P11-24	107	2	2	1	1	5	6	1	1	5	4	3	3	2	4	1	2	4	4	3	3	1
	45P11-26	107	2	2	1	1	5	6	1	1	5	4	3	3	2	4	1	2	4	4	3	3	1
	45P11-28	107	2	2	1	1	5	6	1	1	5	4	3	3	2	4	1	2	4	4	3	3	1
	527	109	2	2	1	1	5	7	1	2	5	4	5	3	2	4	2	2	4	4	3	3	1
	45P11-9	111	2	2	1	1	1	3	1	2	4	4	3	3	2	4	2	2	5	4	3	3	1
	46P11-14	111	2	2	1	1	1	3	1	2	4	4	3	3	2	4	2	2	5	4	3	3	1
	4203	112	5	2	2	3	5	9	2	2	3	4	3	3	2	1	2	2	11	4	3	3	1
	4172	112	5	2	2	3	5	9	2	2	3	4	3	3	2	1	2	2	11	4	3	3	1
	4211	114	5	2	2	3	7	8	2	2	4	6	3	3	1	7	1	2	3	5	3	3	1
	4188	116	5	3	2	3	7	7	2	2	4	6	3	3	1	6	2	2	3	5	3	3	1
	4189	117	2	3	1	4	9	6	2	2	4	6	3	3	1	9	2	2	3	5	3	3	1
	4215	118	5	3	1	3	9	9	2	2	4	6	3	3	1	9	2	2	3	5	3	3	1
	4218	119	5	3	2	3	7	14	2	2	3	6	3	3	1	10	2	2	3	5	3	3	1
	4181	119	5	3	2	3	7	14	2	2	3	6	3	3	1	10	2	2	3	5	3	3	1
	4178	120	5	3	2	3	10	14	1	2	4	6	3	3	1	10	2	2	3	5	3	3	1
	4202	122	5	2	1	1	7	7	2	2	4	6	3	3	2	7	1	2	3	6	3	3	1
	4217	123	2	3	1	3	11	7	2	2	4	2	3	3	1	7	2	2	3	6	3	3	1
	423	124	2	1	2	1	4	3	1	2	5	6	6	3	2	4	2	1	4	6	3	3	1
	45P11-22	127	2	1	3	1	3	7	1	2	4	6	6	3	2	4	2	2	4	3	4	3	1
	45P11-44	128	2	1	3	1	3	7	1	2	5	6	6	3	2	4	2	2	4	3	4	3	1
	4193	136	5	2	1	1	4	10	1	2	4	6	3	3	1	9	1	2	3	4	3	5	1
	4192	137	2	3	1	1	6	7	2	1	4	6	3	3	1	7	2	2	10	4	3	5	1
	CBS_135.94	150	4	3	1	3	9	9	3	3	3	6	4	5	1	8	2	2	3	4	4	4	2
	CBS_113.89	1	4	3	1	5	5	5	2	5	3	4	3	4	1	2	2	2	6	1	1	1	1
	CBS_112.89	7	2	1	2	3	3	7	3	1	5	4	4	3	2	6	2	2	3	8	1	3	1
	LuB8001	10	2	3	2	5	4	5	1	2	5	4	4	3	1	3	1	2	3	1	3	3	1
	SpB6203	22	2	2	2	1	7	3	1	2	4	6	1	3	2	4	2	2	3	3	3	3	1
	SB0501	24	2	1	3	1	7	5	1	2	4	6	4	3	2	4	2	2	3	3	3	3	1
	LuB5101	29	2	1	2	1	6	3	1	2	4	6	5	3	2	4	2	2	3	3	3	3	1
	SpB5101	29	2	1	2	1	6	3	1	2	4	6	5	3	2	4	2	2	3	3	3	3	1
	SpB4901	29	2	1	2	1	6	3	1	2	4	6	5	3	2	4	2	2	3	3	3	3	1
	LuB0501	29	2	1	2	1	6	3	1	2	4	6	5	3	2	4	2	2	3	3	3	3	1
	LuB0601	29	2	1	2	1	6	3	1	2	4	6	5	3	2	4	2	2	3	3	3	3	1
	SpB0401	29	2	1	2	1	6	3	1	2	4	6	5	3	2	4	2	2	3	3	3	3	1
Rat	SpB4801	46	2	1	3	1	6	3	1	2	4	6	6	3	2	4	2	2	4	3	3	3	1
	LuB7401	104	2	1	2	1	4	3	1	2	5	6	6	3	2	4	2	1	4	4	3	3	1
	LuB3801	104	2	1	2	1	4	3	1	2	5	6	6	3	2	4	2	1	4	4	3	3	1
	LuB3701	104	2	1	2	1	4	3	1	2	5	6	6	3	2	4	2	1	4	4	3	3	1
	LuB4001	104	2	1	2	1	4	3	1	2	5	6	6	3	2	4	2	1	4	4	3	3	1
	SpB7001	104	2	1	2	1	4	3	1	2	5	6	6	3	2	4	2	1	4	4	3	3	1
	CBS_111.89	125	2	1	2	3	3	9	1	1	5	4	4	3	1	5	1	2	5	7	3	3	1
	CBS_110.89	126	2	1	2	3	3	6	3	1	5	4	4	3	2	6	2	2	3	8	3	3	1
	CBS_119.89	129	2	1	2	4	4	2	1	1	5	6	4	3	2	4	1	2	4	4	4	3	1
	CBS_109.89	130	2	5	1	1	4	8	1	2	3	6	2	3	1	3	1	2	3	8	4	3	1
	CBS_115.89	131	3	1	2	3	3	6	3	1	5	4	4	3	2	6	2	2	3	8	4	3	1
	CBS_114.89	134	2	3	2	4	5	12	1	1	5	3	4	3	1	3	2	2	2	1	3	4	1

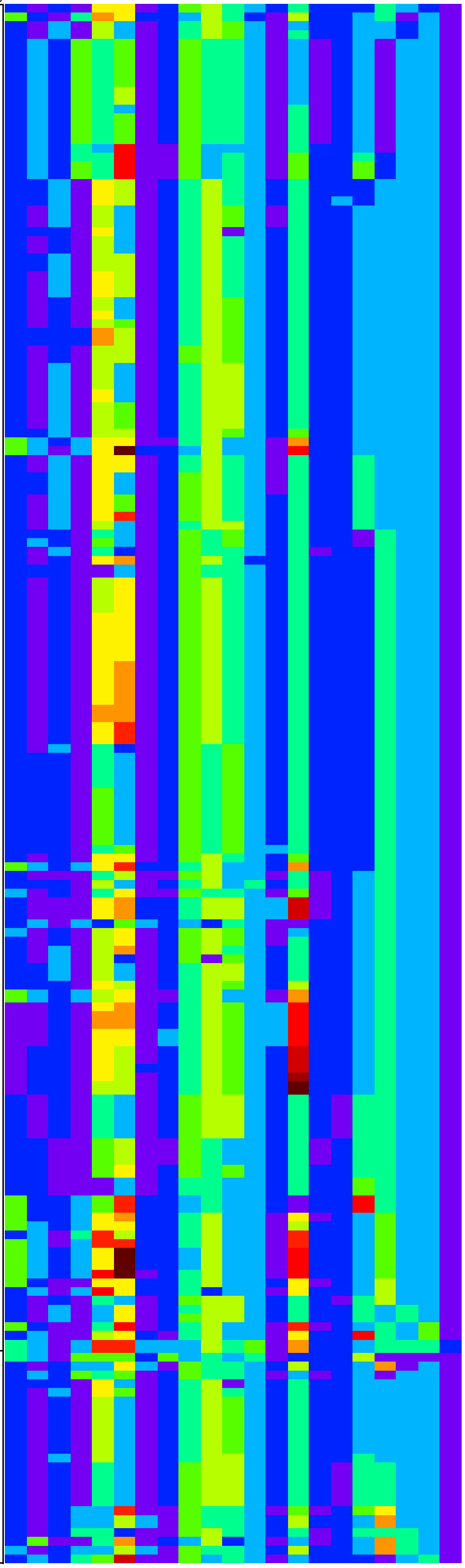

Note: Matrix rainbow-plot showing the alleles present at each locus.
